# Supplementary material for: Design of a Portable Orthogonal Surface Acoustic Wave Sensor System for Simultaneous Sensing and Removal of Nonspecifically Bound Proteins
Source: Sensors (Basel). 2019 Sep 8;19(18):3876. doi: 10.3390/s19183876 (PMC6767010; doi:10.3390/s19183876)
Supplement: Supplementary file 1 [file sensors-19-03876-s001.pdf]

Supplementary Material

The phase vs frequency response of the prototype was compared with the VNA test results, as show in Figure S1. The VNA provides the phase range from -180 to 180 degrees, whereas our prototype provides the phase range from 0 to 180 degrees. Hence, the apparent difference in appearance between the two data.

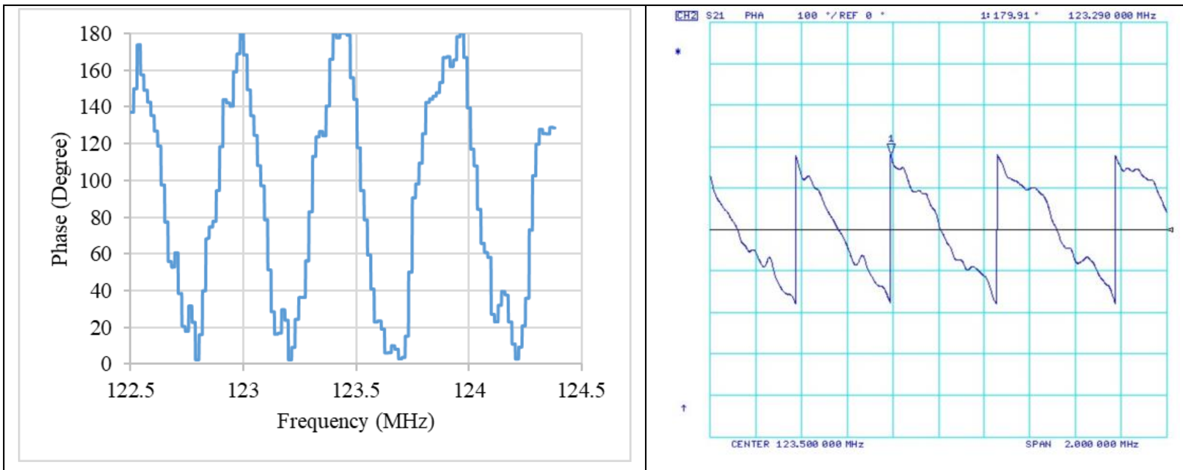

(a) (b)

Figure S1. Comparison of phase vs frequency (a) portable prototype and (b) VNA.
